# Applications of Tris(4-(thiophen-2-yl)phenyl)amine- and Dithienylpyrrole-based Conjugated Copolymers in High-Contrast Electrochromic Devices

**DOI:** 10.3390/polym8060206

**Published:** 2016-05-27

**Authors:** Tzi-Yi Wu, Hsin-Hua Chung

**Affiliations:** Department of Chemical and Materials Engineering, National Yunlin University of Science and Technology, Yunlin 64002, Taiwan; k309798@yahoo.com.tw

**Keywords:** electrochemical polymerization, optical contrast, spectroelectrochemistry, coloration efficiency, electrochromic devices

## Abstract

Tris(4-(thiophen-2-yl)phenyl)amine- and dithienylpyrrole-based copolymers (P(TTPA-*co*-DIT) and P(TTPA-*co*-BDTA)) were electropolymerized on ITO electrode by applying constant potentials of 1.0, 1.1, and 1.2 V. Spectroelectrochemical investigations revealed that P(TTPA-*co*-DIT) film displayed more color changes than P(TTPA-*co*-BDTA) film. The P(TTPA-*co*-DIT) film is yellow in the neutral state, yellowish-green and green in the intermediate state, and blue (1.2 V) in highly oxidized state. The ∆*T*_max_ of the P(TTPA-*co*-DIT) and P(TTPA-*co*-BDTA) films were measured as 60.3% at 1042 nm and 47.1% at 1096 nm, respectively, and the maximum coloration efficiency (η) of P(TTPA-*co*-DIT) and P(TTPA-*co*-BDTA) films were calculated to be 181.9 cm^2^·C^−^^1^ at 1042 nm and 217.8 cm^2^·C^−^^1^ at 1096 nm, respectively, in an ionic liquid solution. Dual type electrochromic devices (ECDs) consisting of P(TTPA-*co*-DIT) (or P(TTPA-*co*-BDTA)) anodic copolymer, ionic liquid-based electrolyte, and poly(3,4-(2,2-diethylpropylenedioxy)thiophene) (PProDOT-Et_2_) cathodic polymer were constructed. P(TTPA-co-BDTA)/PProDOT-Et_2_ ECD showed high Δ*T*_max_ (48.1%) and high coloration efficiency (649.4 cm^2^·C^−^^1^) at 588 nm. Moreover, P(TTPA-*co*-DIT)/PProDOT-Et_2_ and P(TTPA-*co*-BDTA)/PProDOT-Et_2_ ECDs displayed satisfactory optical memory and long term switching stability.

## 1. Introduction

Functional conjugated polymers (CPs) have attracted an increasing attention due to their potentials for unique optical and electrochemical properties, multiple color exhibitions, and high optical contrast ability. In recent years, several kinds of CPs are promising candidates for a large number of advanced technological applications in electrochromic devices (ECDs) [1,2,3], automotive rear-view mirrors [4], light-emitting diodes [5], architectural energy-saving windows [6], catalysts [7,8,9], displays [10], and sensors [11,12]. Especially, CPs-based electrochromic materials, such as polyanilines [13], polycarbazoles [14], polypyrroles [15], polyindoles [16], polythiophenes [17,18,19], poly(3,4-ethylenedioxythiophene)(PEDOT) [20], and polytriphenylamine [21], have been extensively investigated for using as anodic (or cathodic) layers in ECDs. Among these materials, polytriphenylamine possesses hole conducting properties and can be easily oxidized to form polarons, the redox process exhibits obvious color variations. Hsiao *et al.* reported that triphenylaime-containing polyamides displayed good electrochemical stability and multicolor electrochromic behaviors upon applying potentials [22], the percent transmittance change and coloration efficiency of triphenylamine-containing polyamides are 58% and 209 cm^2^·C^−^^1^ at 929 nm, respectively. Polythiophenes and their derivatives are promising organic CPs for commercial electrochromic applications due to their potential to combine with other electrochromic materials to display multichromic behaviors. However, most polythiophenes are insoluble in general solvents and the *E*_onset_ of polythiophenes is high (*ca.* 1.5 V *vs.* Ag/AgCl) [23], the attachment of alkyl and alkoxy groups to polythiophenes gives rise to good solubility and processability of the polymer in general solvents. Moreover, the incorporation of electron donating substituents to the 3- and 4-positions of polythiophenes decreases the *E*_onset_ of polythiophenes. Poly(2,5-dithienylpyrrole)s (PSNS) are one of the promising polythiophenes derivatives due to their low oxidation potential (*ca.* 0.7 V *vs.* Ag/AgCl) and exhibit multichromic behaviors (yellow, green and blue) upon applying potentials. The incorporation of substituents to the nitrogen atom of central pyrrole unit in PSNS backbone gives rise to tunable band gap of PSNS and makes possible modifications to properties. Recently, a number of SNS derivatives have been reported and displayed specific electrochromic properties. Camurlu *et al.* synthesized anthracene- and pyrene-containing thienylpyrrole derivatives (SNS-Anth [24] and SNS-pyrene [25]) and reported their multichromic properties. Koyuncu *et al.* reported the synthesis and electrochromic characterizations of a novel polymer (PSNS-PDI) consisting of SNS-donor and PDI-acceptor units [26]. PSNS-PDI has a high optical contrast (Δ*T* = 45% at 900 nm), a short response speed (0.5 s), and a high coloration efficiency (254 cm^2^·C^−^^1^). Shim *et al.* incorporated 3-pyridinyl and 1,10-phenanthroline units in the central pyrrole ring of PSNS, which were named polyPTPy and polyPhenTPy [27], and the Δ*T* of polyPTPy and polyPhenTPy were 42% and 31%, respectively. Furthermore, copolymers provide an effective method for controlling the properties of CPs, copolymerization is a facile way to combine (or change) the electrochromic properties of individual homopolymers. Toppare *et al.* synthesized a copolymer (P(FPTP-*co*-EDOT)) using FPTP and EDOT electrochemically [28], P(FPTP) has only two colors in its reduced and oxidized states (yellow and blue), while its copolymer with EDOT shows five colors (light gray, green, purple, red, and blue), indicating the incorporation of specific units in copolymer backbone gives rise to multichromic behaviors.

In the present work, two tris(4-(thiophen-2-yl)phenyl)amine- and 2,5-dithienylpyrrole-based conducting copolymers (P(TTPA-*co*-DIT) and P(TTPA-*co*-BDTA)) were prepared using electrochemical copolymerizations. The chemical structure of indene is similar to 1,2,5-thiadiazole, and both of them were introduced to the central pyrrole ring of the SNS unit. The two –C=N– groups in 1,2,5-thiadiazole group increase the electron-withdrawing ability, decrease the lowest unoccupied molecular orbital (LUMO) level, and reduce the band gap of the SNS unit, thereby expand absorbance to longer wavelength region. Moreover, tris(4-(thiophen-2-yl)phenyl)amine (TTPA) unit combines the individual properties of triphenylamine and thiophene moieties. Although the oxidation potential of polythiophenes is *ca.* 1.5 V *vs.* Ag/AgCl [23], the incorporation of a triphenylamine unit among three thiophene groups increase the electron-donating ability of entire TTPA unit. Consequently, poly(tris(4-(thiophen-2-yl)phenyl)amine) shows lower oxidation potential than that of polythiophenes. It was interesting to incorporate the TTPA unit in the copolymer backbone and explore their spectroelectrochemical and electrochromic behaviors. Moreover, the electrochromic devices (ECDs) were constructed using P(TTPA-*co*-DIT) and P(TTPA-*co*-BDTA) as the anodic materials of coloring electrodes, PProDOT-Et_2_ as the cathodic material of complementary electrodes, and an ionic liquid and poly(vinylidene fluoride-*co*-hexafluoropropylene) (PVDF-HFP) composite films as the electrolyte membranes. The spectroelectrochemical properties, such as percent transmittance changes, electrochromic switching, coloration efficiency, and stability of the ECDs were systematically and comprehensively investigated.

## 2. Materials and Methods

### 2.1. Materials and Electrochemical Synthesis

Tris(4-(thiophen-2-yl)phenyl)amine, 1-ethyl-3propylimidazolium bis(trifluoromethanesulfonyl)imide (EPIDIL), SNS derivatives, and ProDOT-Et_2_ were prepared from previously published procedures [29,30,31,32]. P(TTPA-*co*-DIT) and P(TTPA-*co*-BDTA) films were prepared potentiostatically at 1.0, 1.1, and 1.2 V on ITO glass electrodes with an applied charge density of 30 mC·cm^−2^. The ITO is conductive with an electrical resistivity of 10^−4^ Ω·cm. The active area of polymer films on ITO glass was 1.8 cm^2^. Film thicknesses of deposited polymers were obtained with an Alpha-Step profilometer (KLA Tencor D-120, KLA-Tencor, Milpitas, CA, USA). The thicknesses of P(TTPA-*co*-DIT) and P(TTPA-*co*-BDTA) films were about 45 and 50 nm, respectively, for the CV and spectroelectrochemical experiments. P(TTPA-*co*-DIT) and P(TTPA-co-BDTA) films were prepared using feed molar ratio of TTPA/DIT (or TTPA/BDTA) at 1/1.

### 2.2. Construction of ECDs and Spectroelectrochemical Characterizations

Electrolyte membranes were prepared by a casting solution containing PVDF-HFP, EPIDIL, DMF solvent, and propylene carbonate. The weight ratio of PVDF-HFP:EPIDIL:propylene carbonate = 1:3:2. ECDs were constructed using P(TTPA-*co*-DIT) (or P(TTPA-co-BDTA)) as the anodic material and PProDOT-Et_2_ as the cathodic material. The PProDOT-Et_2_ film was electrodeposited onto ITO glass at +1.4 V. ECDs were constructed using anodic and cathodic polymer films facing each other to be separated by an electrolyte membrane.

Electrochemical experiments were carried out in a three-component cell with a CHI660a electrochemical analyzer (CH Instruments, Austin, TX, USA). ITO glass plate, platinum wire, and Ag/AgCl electrode were used as working, counter, and reference electrodes, respectively. Spectroelectrochemical experiments were studied with a V-630 JASCO UV-Visible spectrophotometer (JASCO International Co., Ltd., Tokyo, Japan) to record *in situ* UV-Vis spectra and were done in a quartz cuvette of 1 cm path length assembled as an electrochemical cell with an ITO working electrode, a platinum wire, and an Ag/AgCl reference electrode.

## 3. Results and Discussion

### 3.1. Electrochemical Polymerization

**Figure 1:**
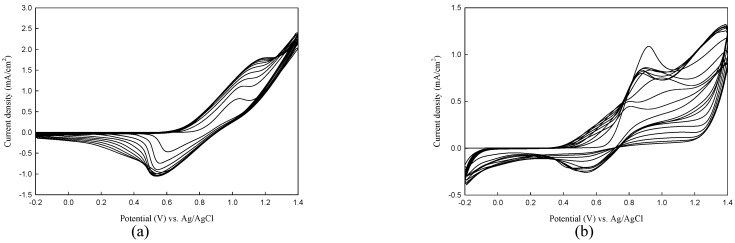

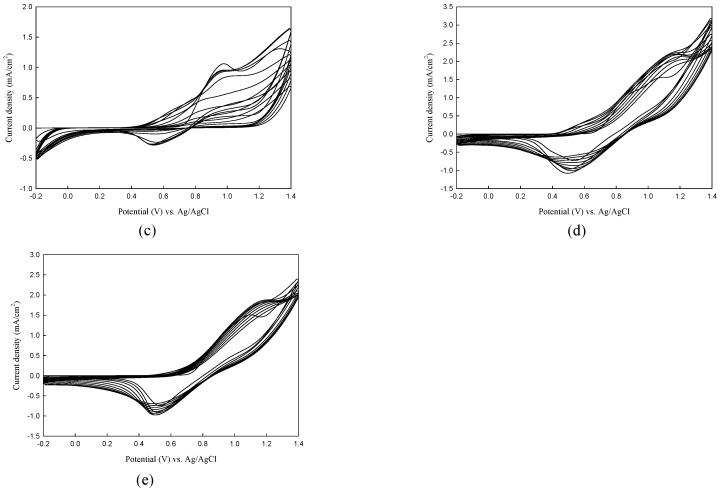
Cyclic voltammograms of: (**a**) 2 mM tris(4-(thiophen-2-yl)phenyl)amine (TTPA); (**b**) 2 mM 1-(2,3-dihydro-inden-4-yl)-2,5-di(thiophen-2-yl)-pyrrole (DIT); (**c**) 2 mM 4-(2,5-di(thiophen-2-yl)-pyrrol-1-yl)benzo[c][1,2,5]thiadiazole (BDTA); (**d**) 2 mM TTPA + 2 mM DIT; and (**e**) 2 mM TTPA + 2 mM BDTA in 0.1 M LiClO_4_/ACN at a scan rate of 100 mV s^−1^ on ITO working electrode.

**Figure 2:**
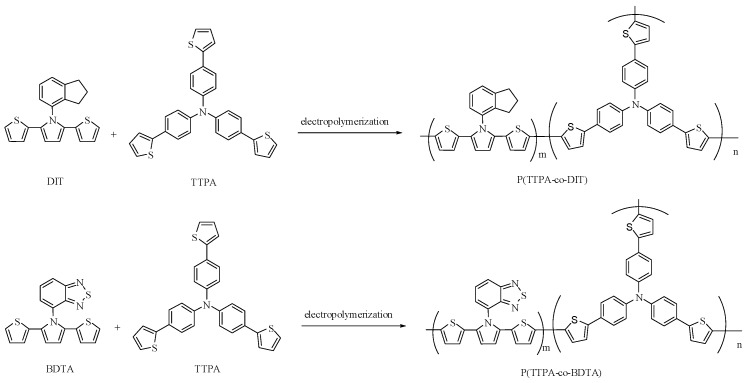
The electrochemical copolymerization of dithienylpyrrole (SNS) derivatives and tris(4-(thiophen-2-yl)phenyl)amine.

The polarization curves of neat TTPA, DIT, and BDTA monomers, and the mixture of two monomers (TTPA + DIT and TTPA + BDTA) in 0.1 M LiClO_4_/ACN are shown in Figure 1. PTTPA showed *E*_onset_ and oxidation peak at 0.79 and 1.16 V, respectively (Figure 1a). On the other hand, the *E*_onset_ of PDIT and PBDTA films were 0.68 and 0.73 V, respectively, and the oxidation peaks of PDIT and PBDTA films were 0.90 and 0.97 V, respectively (Figure 1b,c). When the CVs were scanned in 0.1 M LiClO_4_/ACN solution containing two monomers (2 mM TTPA + 2 mM DIT and 2 mM TTPA + 2 mM BDTA), the CVs’ shape and redox peaks observed in Figure 1d,e are different to those of PTTPA, PDIT, and PBDTA homopolymer films, demonstrating the formation of copolymers. The electrosynthetic routes of P(TTPA-*co*-DIT) and P(TTPA-*co*-BDTA) are shown in Figure 2.

**Figure 3:**
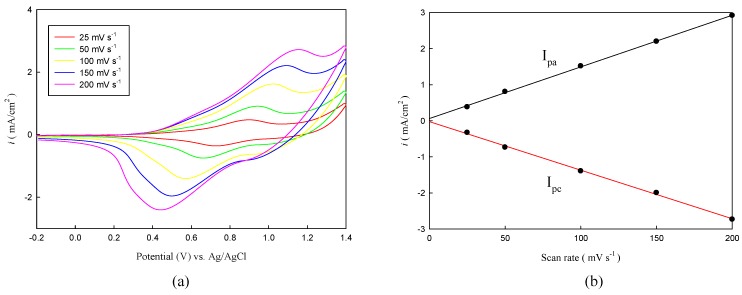
(**a**) CV curves of the P(TTPA-*co*-DIT) film at various scan rates between 25 and 200 mV·s^−1^ in 0.1 M LiClO_4_/ACN solution. P(TTPA-*co*-DIT) film was prepared potentiostatically at 1.0 V; (**b**) Relation between peak current density and scan rate of the P(TTPA-*co*-DIT) film in 0.1 M LiClO_4_/ACN solution.

**Figure 4:**
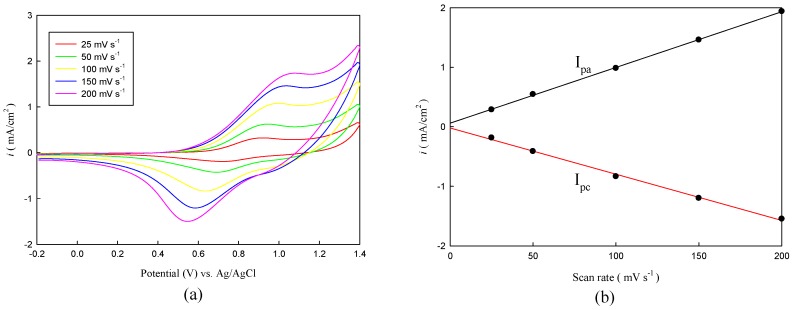
(**a**) CV curves of the P(TTPA-*co*-BDTA) film at various scan rates between 25 and 200 mV·s^−1^ in 0.1 M LiClO_4_/ACN solution. P(TTPA-*co*-BDTA) film was prepared potentiostatically at 1.0 V; (**b**) Relation between peak current density and scan rate of the P(TTPA-*co*-BDTA) film in 0.1 M LiClO_4_/ACN solution.

Copolymer films P(TTPA-*co*-DIT) and P(TTPA-*co*-BDTA) prepared by constant potential deposition at 1.0 V were scanned at different rates in the range from 25 to 200 mV·s^−1^ in 0.1 M LiClO_4_/ACN solution. As can be seen in Figure 3a and Figure 4a, the P(TTPA-*co*-DIT) and P(TTPA-*co*-BDTA) presented two well-defined redox peaks, the current density response increased with the increasing of the scan rate, indicating that the copolymer films had good electrochemical activity and were adhered well to the electrode. With the increasing scan rate, the anodic and cathodic peak current densities showed a linear dependence on the scan rate as illustrated in Figure 3b and Figure 4b, demonstrating that the redox process of the copolymers were not limited by diffusion control [33].

### 3.2. Electrochromic Properties of the Copolymer Films

**Figure 5:**
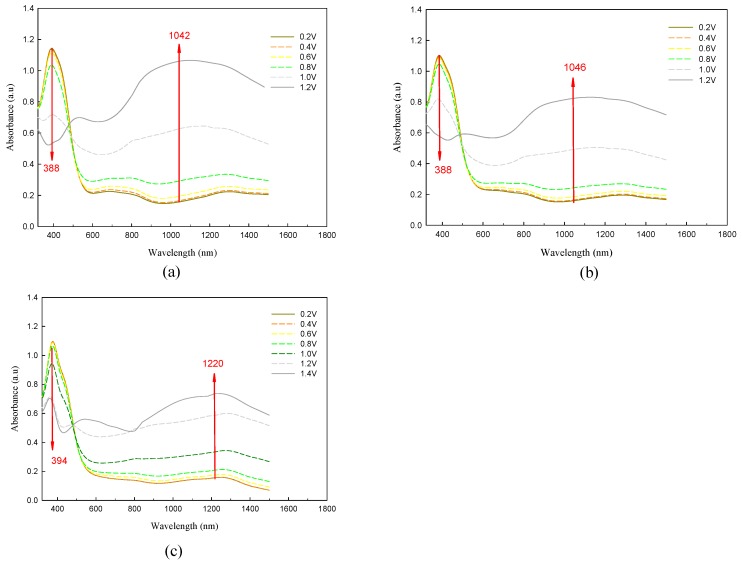
Spectroelectrochemical spectra of P(TTPA-*co*-DIT) films on ITO electrode at various potentials in an ionic liquid solution. The copolymer films were prepared potentiostatically at: (**a**) 1.0 V; (**b**) 1.1 V; and (**c**) 1.2 V on ITO glass electrodes.

**Figure 6:**
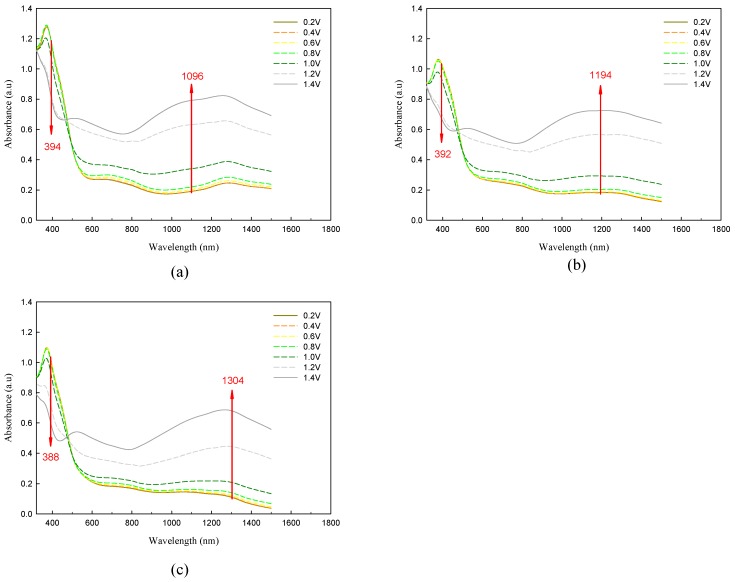
Spectroelectrochemical spectra of P(TTPA-*co*-BDTA) films on ITO electrode as applied potentials between 0.2 V and 1.4 V in an ionic liquid solution. The copolymer films were prepared potentiostatically at: (**a**) 1.0 V; (**b**) 1.1 V; and (**c**) 1.2 V on ITO glass electrodes.

Spectroelectrochemistry combines electrochemical and spectroscopic methods for investigating the changes in the absorption spectra upon applying of an external electrical potential. Spectroelectrochemistry of P(TTPA-*co*-DIT) and P(TTPA-*co*-BDTA) copolymer films coated on ITO electrode was studied in an ionic liquid solution. Figure 5 displayed the spectroelectrochemical spectra of P(TTPA-*co*-DIT) film at various potentials in EPIDIL solution. The copolymer films were prepared potentiostatically at 1.0 V, 1.1 V, and 1.2 V (see Figure 5a–c, respectively). As shown in Figure 5a and Table 1, the peak of P(TTPA-*co*-DIT) film in the neutral state was found at 388 nm, which corresponded to the π–π * transition of P(TTPA-*co*-DIT) in EPIDIL solution. Upon applying more than 0.8 V, the absorbance of π–π * transition peak of P(TTPA-*co*-DIT) decreased gradually and charge carrier bands appeared in higher wavelength region, which corresponded to the development of polaron and bipolaron bands [34]. When the P(TTPA-*co*-DIT) film was prepared potentiostatically at 1.1 V and 1.2 V, the π–π * transition of P(TTPA-*co*-DIT) film did not shift significantly. However, the position of polaron peak with maximal absorbance changes shifted conspicuously upon applying various potentials, this can be ascribed to adherent polymer films undergo configuration changes during electrochemical overoxidation [35]. The π–π * transition of P(TTPA-*co*-BDTA) film in EPIDIL solution located at similar position with P(TTPA-*co*-DIT) film, whereas the polaron peak positions of P(TTPA-*co*-BDTA) film with maximal absorbance shifted bathochromically relative to those of P(TTPA-*co*-DIT) film upon applying various potentials (Figure 6), which could be attributed to an electron-withdrawing 1,2,5-thiadiazole unit in BDTA unit showed narrower band gap in EPIDIL solution than that of DIT unit.

Table 2 shows the photographs and colorimetric values (L*, a*, b*) of the copolymer films at various potentials in EPIDIL solution. The P(TTPA-*co*-DIT) film was yellow (0.2 V) in the neutral state, yellowish-green (0.8 V) and green (1.0 V) in the intermediate state, and blue (1.2 V) in highly oxidized state. The P(TTPA-*co*-BDTA) film showed less color changes than those of P(TTPA-*co*-DIT) film, P(TTPA-*co*-BDTA) film was yellow (0.2 V) in the neutral state, bluish-green (1.0 V) in the intermediate state, and blue (1.2 V) in highly oxidized state, indicating the incorporation of DIT unit into copolymer backbone gives rise to more color changes than that of BDTA unit.

**Figure 7:**
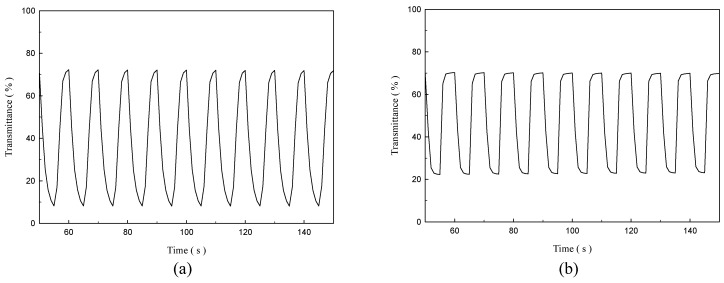
*In situ* transmittance of: (**a**) P(TTPA-*co*-DIT); and (**b**) P(TTPA-*co*-BDTA) films as a function of time in EPIDIL electrolyte, the time interval is 5 s. The copolymer films were prepared potentiostatically at 1.0 V on ITO electrodes and were stepped by repeated potential between 0.2 V and +1.2 V.

A square-wave potential step technology coupled with a UV-Visible spectrophotometer was used for analysis of switching kinetics and optical contrast of the copolymer films [36]. The P(TTPA-*co*-DIT) and P(TTPA-*co*-BDTA) films were stepped by repeated potential between neutral state (0.2 V) and oxidized state (+1.2 V) with a time interval of 5 s in an ionic liquid solution. The *in situ* transmittance–time profiles of P(TTPA-*co*-DIT) and P(TTPA-*co*-BDTA) films in EPIDIL solution are displayed in Figure 7, and the optical contrast (∆*T*) estimated at 1st, 50th, and 100th cycles are summarized in Table 3. For P(TTPA-*co*-DIT) film prepared potentiostatically at 1.0 V, 1.1 V, and 1.2 V, the ∆*T* of P(TTPA-*co*-DIT)-1.0 V, P(TTPA-*co*-DIT)-1.1 V, and P(TTPA-*co*-DIT)-1.2 V films at first cycle are 60.3, 55.6, and 49.4%, respectively, and P(TTPA-*co*-DIT) film prepared potentiostatically at 1.0 V shows the highest ∆*T*. For the Δ*T* of copolymer films at different switching cycles, the Δ*T* of P(TTPA-*co*-DIT)-1.0 V film from the bleaching state to the coloration state in EPIDIL solution was 60.3, 58.8 and 57.1%, respectively, at 1st, 50th, and 100th cycle. However, the Δ*T* of P(TTPA-*co*-DIT)-1.2 V film from the bleaching state to the coloration state in EPIDIL solution was 49.4, 43.2 and 42.6%, respectively, at 1st, 50th, and 100th cycle. The stability of P(TTPA-*co*-DIT)-1.0 V and P(TTPA-*co*-DIT)-1.2 V films at the 100th cycle was 94.7 and 86.2%, respectively, and the P(TTPA-*co*-DIT)-1.0 V film shows higher stability than that of P(TTPA-*co*-DIT)-1.2 V film at high switching cycles, which can be attributed to an overoxidation of the copolymer takes place when electropolymerization at high potential (*i.e.*, in highly oxidized state). The Δ*T* of P(TTPA-*co*-DIT)-1.0 V and P(TTPA-*co*-BDTA)-1.0 V films from the bleaching state to the coloration state in EPIDIL solution were 60.3 and 47.1%, respectively, at the first cycle, implying P(TTPA-*co*-DIT) film shows higher Δ*T* than that of P(TTPA-*co*-BDTA) film. The stability of P(TTPA-*co*-DIT)-1.0 V and P(TTPA-*co*-BDTA)-1.0 V films at the 100^th^ cycle was 94.7 and 85.6%, respectively, revealing the P(TTPA-*co*-DIT) film shows higher stability than that of P(TTPA-*co*-BDTA) film at high switching cycles.

The coloration switching time (τ_c_) and the bleaching switching time (τ_b_) of copolymer films estimated at 1st, 50th, and 100th cycles are also summarized in Table 3. The switching time was estimated at 90% of the full-transmittance variation. P(TTPA-*co*-BDTA) film shows shorter τ_c_ and τ_b_ than those of P(TTPA-*co*-DIT) film, revealing that P(TTPA-*co*-BDTA) film exhibits fast switching speeds from the dedoped to the doped state and from the doped to the dedoped state when we employ EPIDIL as a supporting electrolyte. The Δ*T*_max_ of P(TTPA-*co*-DIT)-1.0 V and P(TTPA-*co*-BDTA)-1.0 V films are higher than that reported for PTTPA derivative (P(TTPA-*co*-EDOT)) [37], and higher than those reported for PSNS derivatives (PTEPA [38], PSNS-1-NAPH [39], and P(SNS-Fc-*co*-EDOT) [40]). This could be ascribed to the fact that Δ*T*_max_ of P(TTPA-*co*-DIT)-1.0 V and P(TTPA-*co*-BDTA)-1.0 V films were estimated in long wavelength region (1042–1096 nm) when we employed EPIDIL as a supporting electrolyte.

ΔOD is the discrepancy of optical density, which can be estimated using the transmittance of the oxidation state (*T*_ox_) and neutral state (*T*_neu_) using the following equation:

(1) $$\Delta \mathrm{OD}=\log\left(\frac{T_{\mathrm{ox}}}{T_{\mathrm{neu}}}\right)$$

The ΔOD_max_ of P(TTPA-*co*-DIT)-1.0 V film at 1042 nm and P(TTPA-*co*-BDTA)-1.0 V film at 1096 nm in EPIDIL solution are 80 and 49%, respectively. Similar to Δ*T*_max_, P(TTPA-*co*-DIT)-1.0 V film showed higher ΔOD_max_ than that of P(TTPA-*co*-BDTA)-1.0 V film.

The coloration efficiency (η) at a specific wavelength can be defined as the ΔOD for the charge (*q*) consumed per unit electrode area (*A*):

(2) $$\eta =\frac{\Delta \mathrm{OD}}{q/A}$$

As shown in Table 4, the η_max_ of P(TTPA-*co*-DIT)-1.0 V film at 1042 nm and P(TTPA-*co*-BDTA)-1.0 V film at 1096 nm in EPIDIL solution are 181.9 and 217.8 cm·C^−1^, respectively, which were higher than those reported for PTEPA [38] at 448 nm and PSNS-1-NAPH [39] at 423 nm.

### 3.3. Spectroelectrochemistry of ECDs

**Figure 8:**
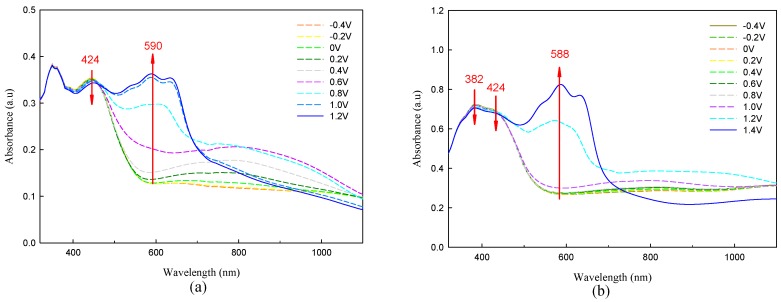
Spectroelectrochemical spectra of: (**a**) P(TTPA-*co*-DIT)/PProDOT-Et_2_; and (**b**) P(TTPA-co-BDTA)/PProDOT-Et_2_ ECDs as applied potentials between −0.4 V and 1.2 V. P(TTPA-*co*-DIT) and P(TTPA-*co*-BDTA) films were prepared potentiostatically at 1.0 V.

Dual type ECDs consisting of electrochemically deposited P(TTPA-*co*-DIT)-1.0 V/PProDOT-Et_2_ and P(TTPA-*co*-BDTA)-1.0 V/PProDOT-Et_2_ were constructed and their spectroelectrochemical behaviors were studied by recording the optical absorbance spectra at various potentials. ECDs showed a reversible response in a potential range of −0.4 V and 1.2 V, as depicted in Figure 8. At −0.4 V, P(TTPA-*co*-DIT)-1.0 V/PProDOT-Et_2_ and P(TTPA-*co*-BDTA)-1.0 V/PProDOT-Et_2_ ECDs revealed well defined transitions at *ca.* 382 and 424 nm, respectively, which are in accordance with the spectral behaviors of P(TTPA-*co*-DIT)-1.0 V and P(TTPA-*co*-BDTA)-1.0 V films in reduced state. However, in this situation, the complementary PProDOT-Et_2_ layer is expected to be in oxidized state and it does not show significant transition in UV spectrum. Upon increasing the potential gradually, P(TTPA-*co*-DIT)-1.0 V and P(TTPA-*co*-BDTA)-1.0 V films begin to oxidize and a new absorption band at 588 nm appeared due to neutralization of the PProDOT-Et_2_ layer, and the ECDs were blue in the potential range of +0.8 and 1.2 V for P(TTPA-*co*-DIT)-1.0 V/PProDOT-Et_2_ ECD and in the potential range of +1.0 and 1.4 V for P(TTPA-*co*-BDTA)-1.0 V/PProDOT-Et_2_ ECD (Table 5).

**Figure 9:**
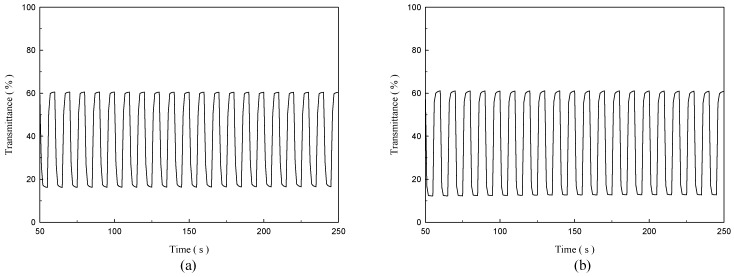
*In situ* transmittance of: (**a**) P(TTPA-*co*-DIT)/PProDOT-Et_2_ ECD (590 nm); and (**b**) P(TTPA-*co*-BDTA)/PProDOT-Et_2_ ECD (588 nm) as a function of time, the time interval is 5 s. The ECDs were stepped by repeated potential between −0.2 V and +1.2 V.

The transmittance–time profiles of P(TTPA-*co*-DIT)-1.0 V/PProDOT-Et_2_ and P(TTPA-*co*-BDTA)-1.0 V/PProDOT-Et_2_ ECDs were shown in Figure 9, which were stepped by repeated potential in the range of neutral (− 0.2 V) and oxidized states (+1.2 V) with a time interval of 5 s, and the Δ*T*, τ_c_, and τ_b_ estimated at different double-step potential cycles are summarized in Table 6. The Δ*T* of P(TTPA-*co*-DIT)-1.0 V/PProDOT-Et_2_ and P(TTPA-*co*-BDTA)-1.0 V/PProDOT-Et_2_ ECDs is 43.5 and 48.1% at the first cycle, respectively, implying P(TTPA-*co*-BDTA) film is a promising electrochromic material to increase the Δ*T* when we employ P(TTPA-*co*-BDTA) film as anodic copolymer layer in ECDs. For P(TTPA-*co*-BDTA) film was prepared potentiostatically at 1.0 V, 1.1 V, and 1.2 V, the ∆*T* of P(TTPA-*co*-BDTA)-1.0 V/PProDOT-Et_2_, P(TTPA-*co*-BDTA)-1.1 V/PProDOT-Et_2_, and P(TTPA-*co*-BDTA)-1.2 V/PProDOT-Et_2_ ECDs at first cycle are 48.1, 35.6, and 30.1%, respectively, and P(TTPA-*co*-BDTA) film prepared potentiostatically at 1.0 V shows the highest ∆*T*. The stability of P(TTPA-*co*-DIT)-1.0 V/PProDOT-Et_2_ and P(TTPA-*co*-DIT)-1.2 V/PProDOT-Et_2_ ECDs at the 100th cycle was 96.3 and 90.8%, respectively, indicating the P(TTPA-*co*-DIT)-1.0 V/PProDOT-Et_2_ ECD shows higher stability than that of P(TTPA-*co*-DIT)-1.2 V/PProDOT-Et_2_ ECD at high switching cycles. Moreover, the stability of P(TTPA-*co*-BDTA)-1.0 V/PProDOT-Et_2_ ECD is higher than P(TTPA-*co*-DIT)-1.0 V/PProDOT-Et_2_ ECD, and the stabilities of ECDs are high than those of copolymer films in an ionic liquid solution. For the optical switching time of P(TTPA-*co*-DIT)/PProDOT-Et_2_ and P(TTPA-*co*-BDTA)/PProDOT-Et_2_ ECDs, the *τ*_c_ and *τ*_b_ of the ECDs were shorter than those of copolymer films in EPIDIL solution, displaying the ECDs changed color faster upon applying potentials than the copolymer films in EPIDIL solution.

Table 7 shows the Δ*T*_max_, ΔOD_max_, and *η*_max_ of P(TTPA-*co*-DIT)/PProDOT-Et_2_ and P(TTPA-*co*-BDTA)/PProDOT-Et_2_ ECDs and reported dual-type ECDs, P(TTPA-*co*-DIT)/PProDOT-Et_2_ and P(TTPA-*co*-BDTA)/PProDOT-Et_2_ ECDs show higher Δ*T*_max_ than those reported for P(SNS-HE)/PEDOT [41], PTEPA/PEDOT [38], P(TTPA-*co*-BT)/PEDOT [42], P(TTPA-*co*-EDOT)/PEDOT [37], and P(Cz4-*co*-CIn1)/PProDOT-Me_2_ [43] ECDs. In another aspect, P(TTPA-*co*-BDTA)/PProDOT-Et_2_ ECD shows higher *η* than that of P(TTPA-*co*-DIT)/PProDOT-Et_2_ ECD. P(TTPA-*co*-BDTA)/PProDOT-Et_2_ ECD shows higher *η* than those reported for PTEPA/PEDOT [38], P(TTPA-*co*-EDOT)/PEDOT [37], and P(Cz4-*co*-CIn1)/PProDOT-Me_2_ ECDs [43], whereas P(TTPA-*co*-BDTA)/PProDOT-Et_2_ ECD shows lower η than that reported for P(SNS-HE)/PEDOT [41] ECD.

### 3.4. Open Circuit Memory of ECDs

**Figure 10:**
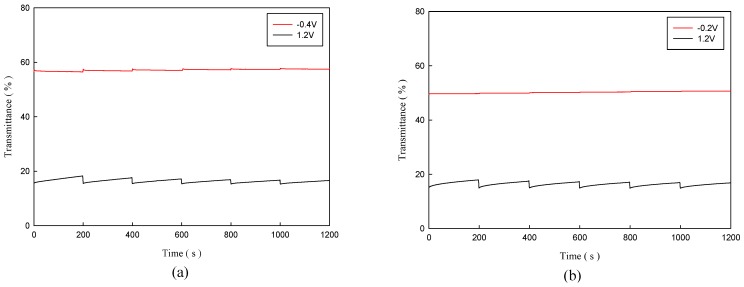
Open circuit stability of the: (**a**) P(TTPA-*co*-DIT)-1.0 V/PProDOT-Et_2_ ECD monitored at 590 nm; and (**b**) P(TTPA-*co*-BDTA)-1.0 V/PProDOT-Et_2_ ECD monitored at 588 nm.

The open circuit memory test of P(TTPA-*co*-DIT)-1.0 V/PProDOT-Et_2_ and P(TTPA-*co*-BDTA)-1.0 V/PProDOT-Et_2_ ECDs were monitored at 590 and 588 nm, respectively, as a function of time by applying potential for 1 s for each 200 s time interval. The test potentials for P(TTPA-*co*-DIT)-1.0 V/PProDOT-Et_2_ ECD were −0.4 and 1.2 V in neutral and oxidized states, respectively, for P(TTPA-*co*-BDTA)-1.0 V/PProDOT-Et_2_ ECD were −0.2 and 1.2 V in neutral and oxidized states, respectively. It can be seen in Figure 10a,b that these ECDs show less than 5% transmittance change in oxidized state and less than 2% transmittance change in neutral state, indicating the presence of good optical memories for the ECDs.

### 3.5. Stability of ECDs

**Figure 11:**
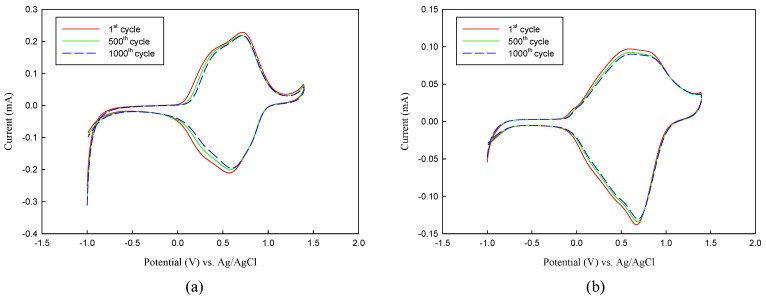
Cyclic voltammogram of: (**a**) P(TTPA-*co*-DIT)-1.0 V/PProDOT-Et_2_ ECD; and (**b**) P(TTPA-*co*-BDTA)-1.0 V/PProDOT-Et_2_ ECD as a function of repeated scans at 100 mV·s^−1^.

Stability upon repeatedly applied potentials between neutral and oxidized states is a way to estimate the electrochromic lifetime of ECDs [44]. Stabilities of the P(TTPA-*co*-DIT)-1.0 V/PProDOT-Et_2_ and P(TTPA-*co*-BDTA)-1.0 V/PProDOT-Et_2_ ECDs were verified by 1000 cycles CV measurements of the applied potential between −1.0 V and 1.4 V with 100 mV·s^−^^1^ scan rate. As shown in Figure 11a,b, the electrochromic switch between neutral and oxidized states of P(TTPA-*co*-DIT)-1.0 V/PProDOT-Et_2_ and P(TTPA-*co*-BDTA)-1.0 V/PProDOT-Et_2_ ECDs, 92% and 96%, respectively, of electroactivity was maintained after 500 cycles, and 88% and 93%, respectively, of electroactivity was retained after 1000 cycles, implying these ECDs exhibited reasonable environmental and redox stability after 1000 cycles.

## 4. Conclusions

Copolymers based on tris(4-(thiophen-2-yl)phenyl)amine and SNS derivatives were electrochemically synthesized and characterized in an ionic liquid solution. Spectroelectrochemical studies exhibited that P(TTPA-*co*-DIT) and P(TTPA-*co*-BDTA) films have distinct electrochromic behaviors from neutral state (yellow) to the oxidized state (blue) upon applying various potentials. Electrochromic switching characterizations of copolymer films indicate that P(TTPA-*co*-DIT)-1.0 V film has higher Δ*T*_max_ (60.3% at 1042 nm) than the P(TTPA-*co*-BDTA)-1.0 V film (47.1% at 1096 nm), whereas P(TTPA-*co*-BDTA)-1.0 V film has higher η_max_ (217.8 cm^2^·C^−1^ at 1096 nm) than the P(TTPA-*co*-DIT)-1.0 V film (181.9 cm^2^·C^−1^ at 1042 nm) in an ionic liquid solution. Dual type ECDs employing P(TTPA-*co*-DIT) (or P(TTPA-*co*-BDTA)) as anodic layer and PProDOT-Et_2_ as cathodic layer were fabricated. The stability of P(TTPA-*co*-BDTA)-1.0 V/PProDOT-Et_2_ ECD was higher than P(TTPA-*co*-DIT)-1.0 V/PProDOT-Et_2_ ECD, and the stabilities of ECDs were higher than those of copolymer films in an ionic liquid solution. The Δ*T*_max_ and η_max_ of P(TTPA-*co*-BDTA)-1.0 V/PProDOT-Et_2_ ECD are 48.1% and 649.4 cm^2^·C^−^^1^ at 588 nm, respectively, which are higher than those of P(TTPA-*co*-DIT)-1.0 V/PProDOT-Et_2_ ECDs. In addition, the stabilities for the color-bleach switching of ECDs at 100th cycles are higher than those of copolymer films characterized in an ionic liquid solution. In view of the above results, the P(TTPA-*co*-DIT)-1.0 V and P(TTPA-*co*-BDTA) films could be useful as the active layers in ECDs.

## Figures and Tables

**Table 1:** Absorption peaks of polymer films in ionic liquid solution.

Polymer films	λ_(π-π* peak)_/nm	λ_(polaron peak)_/nm
P(TTPA-*co*-DIT)-1.0 V	388	1,042
P(TTPA-*co*-DIT)-1.1 V	388	1,046
P(TTPA-*co*-DIT)-1.2 V	394	1,220
P(TTPA-*co*-BDTA)-1.0 V	394	1,096
P(TTPA-*co*-BDTA)-1.1 V	392	1,194
P(TTPA-*co*-BDTA)-1.2 V	388	1,304

**Table 2:** Electrochromic photographs and colorimetric values (L*, a*, b*) of the copolymer films at various potentials in ionic liquid solution.

Polymer films	*E*/V	Photographs	L*	a*	b*
P(TTPA-*co*-DIT)-1.0 V	0.2	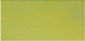	77.29	−4.24	56.58
	0.8	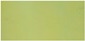	73.53	−6.66	43.36
	1.0	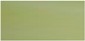	63.02	−1.3	14.59
	1.2	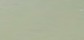	52.53	5.36	−7.73
P(TTPA-*co*-BDTA)-1.0 V	0.2	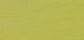	74.32	−5.14	45.18
	1.0	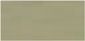	68.96	−5.45	29.7
	1.2	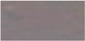	57.32	0.71	6.57
	1.4	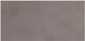	54.12	2.65	1.14

**Table 3:** Electrochromic photographs and colorimetric values (L*, a*, b*) of the copolymer films at various potentials in ionic liquid solution.

Polymer films	λ/nm	Cycle No.	Optical contrast/%			τ/s		Stability (100 cyc)
			*T*_b_	*T*_c_	*ΔT*	τ_c_	τ_b_	
P(TTPA-*co*-DIT)-1.0 V	1042	1	70.9	10.6	60.3	2.64	2.42	
		50	69.5	10.7	58.8	2.48	2.31	
		100	68	10.9	57.1	2.37	2.23	94.7
P(TTPA-*co*-DIT)-1.1 V	1046	1	68.3	12.7	55.6	2.45	2.27	
		50	65.4	13	52.4	2.16	2.67	
		100	65	13.3	51.7	2.11	2.54	93
P(TTPA-*co*-DIT)-1.2 V	1220	1	63.9	14.5	49.4	2.07	2.35	
		50	62.7	19.5	43.2	1.81	1.74	
		100	62.4	19.8	42.6	1.79	1.58	86.2
P(TTPA-*co*-BDTA)-1.0 V	1096	1	69.8	22.7	47.1	1.82	0.99	
		50	68.9	25.9	43	1.64	0.92	
		100	68.3	28	40.3	1.58	0.88	85.6
P(TTPA-co-BDTA)-1.1 V	1194	1	72.1	28.9	43.2	2.15	1.72	
		50	70.9	36.6	34.3	2.07	1.93	
		100	68.6	41.5	27.1	2.32	2.36	62.7
P(TTPA-co-BDTA)-1.2 V	1304	1	78.3	35.7	42.6	1.56	1.61	
		50	77.1	47.1	30	1.22	1.65	
		100	74.5	53.9	20.6	1.26	1.23	48.4

**Table 4:** Summary of Δ*T*_max_ and η_max_ for various polymer films.

Polymer films	Electrolyte	*λ*/nm	Δ*T*_max_/%	∆OD_max_/%	η_max_/cm^2^·C^−1^	Ref.
P(TTPA-*co*-EDOT)	0.2 M NaClO_4_/ACN/DCM	460	30.6	-	-	[37]
P(TTPA-*co*-EDOT)	0.2 M NaClO_4_/ACN/DCM	800	45	-	-	[37]
PTEPA	0.2 M NaClO_4_/ACN/DCM	448	26.84	-	152	[38]
PSNS-1-NAPH	0.1 M LiClO_4_/ethanol	423	18.2	25	102	[39]
P(SNS-Fc-*co*-EDOT)	LiClO_4_/ACN	415	20.9	-	-	[40]
P(TTPA-*co*-DIT)-1.0 V	EPIDIL	1042	60.3	80	181.9	This work
P(TTPA-*co*-BDTA)-1.0 V	EPIDIL	1096	47.1	49	217.8	This work

**Table 5:** Electrochromic photographs and colorimetric values (L*, a*, and b*) of the ECDs. The P(TTPA-*co*-DIT) and P(TTPA-*co*-BDTA) films were prepared potentiostatically at 1.0 V on ITO glass electrodes.

ECDs	*E*/V	Photographs	L*	a*	b*
P(TTPA-*co*-DIT)/PProDOT-Et_2_	−0.4	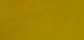	85.82	2.95	32.99
	0.2	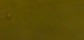	84.21	0.62	29.04
	0.4	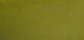	81.66	1.2	24.51
	0.6	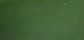	78.26	0.54	18.34
	0.8	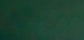	74.76	−0.04	12.22
	1.0	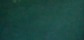	73.54	−0.09	9.24
	1.2	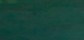	73.06	−0.18	7.31
P(TTPA-*co*-BDTA)/PProDOT-Et_2_	−0.4	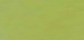	75.43	−5.99	31.89
	0.6	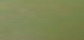	75.57	−6.16	31.99
	0.8	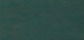	75.46	−6.27	31.78
	1.0	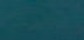	73.87	−5.83	29.33
	1.2	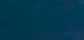	56.54	0.21	4.25
	1.4	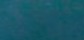	50.25	−2.73	−6.74

**Table 6:** Color–bleach switching time of ECDs.

ECDs	λ/nm	Cycle No.	Optical contrast/%			τ/s		Stability (100 cyc)
			*T*_b_	*T*_c_	*ΔT*	τ_c_	τ_b_	
P(TTPA-*co*-DIT)-1.0 V/ProDOT-Et_2_	590	1	60	16.5	43.5	1.52	1.52	
		50	59.8	17.3	42.5	1.61	1.48	
		100	59.5	17.6	41.9	1.61	1.55	96.3%
P(TTPA-*co*-DIT)-1.1 V/PProDOT-Et_2_	590	1	54.9	20.8	34.1	0.91	0.83	
		50	54.6	21	33.6	0.91	0.87	
		100	54.1	21.3	32.8	0.9	0.86	96.2%
P(TTPA-*co*-DIT)-1.2 V/PProDOT-Et_2_	590	1	54.8	24.2	30.6	0.86	0.85	
		50	54.2	24.3	30	0.83	0.84	
		100	52.4	24.6	27.8	0.82	0.88	90.8%
P(TTPA-*co*-BDTA)-1.0 V/PProDOT-Et_2_	588	1	60.5	12.4	48.1	0.93	0.91	
		50	60.2	13	47.2	0.96	0.97	
		100	59.7	13	46.7	1.04	1.08	97.1%
P(TTPA-*co*-BDTA)-1.1 V/PProDOT-Et_2_	590	1	58.7	23.1	35.6	1.82	1.59	
		50	58.4	23.5	34.2	1.69	1.54	
		100	58.2	23.7	34.5	1.67	1.51	96.9%
P(TTPA-*co*-BDTA)-1.2 V/PProDOT-Et_2_	592	1	58.6	28.5	30.1	1.82	1.4	
		50	58.4	28.7	29.7	1.31	1.21	
		100	58	28.9	29.1	0.86	0.83	96.7%

**Table 7:** The optical contrast, changes of the optical density and coloration efficiency of ECDs.

ECDs	λ/nm	∆*T*_max_/%	∆OD_max_/%	η of ∆OD_max_/cm^2^·C^−1^	Ref.
P(SNS-HE)/PEDOT	570	14.1	-	741	[41]
PTEPA/PEDOT	628	24.72	-	277	[38]
P(TTPA-*co*-BT)/PEDOT	630	30.58	-	-	[42]
P(TTPA-*co*-EDOT)/PEDOT	650	24	-	545	[37]
P(Cz4-*co*-CIn1)/PProDOT-Me_2_	575	32	24.6	372.7	[43]
P(TTPA-*co*-DIT)-1.0 V /PProDOT-Et_2_	590	43.5	56	373.3	This work
P(TTPA-*co*-BDTA)-1.0 V /PProDOT-Et_2_	588	48.1	69	649.4	This work

## References

[1] Abidin T., Zhang Q., Wang K.L., Liaw D.J. (2014). Recent advances in electrochromic polymers. Polymer.

[2] Ak M., Ak M.S., Kurtay G., Güllü M., Toppare L. (2010). Synthesis and electropolymerization of 1,2-bis(thiophen-3-ylmethoxy)benzene and its electrochromic properties and electrochromic device application. Solid State Sci..

[3] Wu T.Y., Su Y.S. (2015). Electrochemical synthesis and characterization of a 1,4-benzodioxan-based electrochromic polymer and its application in electrochromic devices. J. Electrochem. Soc..

[4] De Paoli M., Gazotti W. (2002). Electrochemistry, polymers and opto-electronic devices: A combination with a future. J. Braz. Chem. Soc..

[5] Wu T.Y., Chen Y. (2002). Synthesis, optical and electrochemical properties of novel copolymers containing alternate 2,3-quinoxaline and hole-transporting units. J. Polym. Sci. Part A Polym. Chem..

[6] Xu C., Liu L., Legenski S.E., Ning D., Taya M. (2004). Switchable window based on electrochromic polymers. J. Mater. Res..

[7] Kuo C.W., Chen B.K., Tseng Y.H., Hsieh T.H., Ho K.S., Wu T.Y., Chen H.R. (2012). A comparative study of poly(acrylic acid) and poly(styrenesulfonic acid) doped into polyaniline as platinum catalyst support for methanol electro-oxidation. J. Taiwan Inst. Chem. Eng..

[8] Kuo C.W., Kuo Z.Y., Jow J.J., Wu T.Y., Chen J.Y., Zhu X.X. (2012). Enhanced electrocatalytic performance for methanol oxidation via insertion of ruthenium oxide particles into Pt and polyaniline-poly(acrylic acid-*co*-maleic acid) composite electrode. Int. J. Electrochem. Sci..

[9] Yang C.C., Wu T.Y., Chen H.R., Hsieh T.H., Ho K.S., Kuo C.W. (2011). Platinum particles embedded into nanowires of polyaniline doped with poly(acrylic acid-*co*-maleic acid) as electrocatalyst for methanol oxidation. Int. J. Electrochem. Sci..

[10] Karzazi Y. (2014). Organic light emitting diodes: Devices and applications. J. Mater. Environ. Sci..

[11] Wu T.Y., Sheu R.B., Chen Y. (2004). Synthesis, optically acid-sensory and electrochemical properties of novel polyoxadiazole derivatives. Macromolecules.

[12] Wu T.Y., Chen Y. (2004). Poly(phenylene vinylene)-based copolymers containing 3,8-iminodibenzyl, 3,7-phenothiazinyl and 2,6-pyridinyl chromopjores: Synthesis and fluorescence sensor for acid, metal ion and oxidation. J. Polym. Sci. Part A Polym. Chem..

[13] Kuo C.W., Chen B.K., Li W.B., Tseng L.Y., Wu T.Y., Tseng C.G., Chen H.R., Huang Y.C. (2014). Effects of supporting electrolytes on spectroelectrochemical and electrochromic properties of polyaniline-poly(styrene sulfonic acid) and poly(ethylenedioxythiophene)-poly(styrene sulfonic acid)-based electrochromic device. J. Chin. Chem. Soc..

[14] Sefer E., Koyuncu F.B., Oguzhan E., Koyuncu S. (2010). A new near-infrared switchable electrochromic polymer and its device application. J. Polym. Sci. Part A Polym. Chem..

[15] Mert O., Demir A.S., Cihaner A. (2013). Pyrrole coupling chemistry: investigation of electroanalytic, spectroscopic and thermal properties of N-substituted poly(bis-pyrrole) films. RSC Adv..

[16] Nie G., Qu L., Xu J., Zhang S. (2008). Electrosyntheses and characterizations of a new soluble conducting copolymer of 5-cyanoindole and 3,4-ethylenedioxythiophene. Electrochim. Acta.

[17] Silva A.J.C., Ferreira S.M.F., Santos D.P., Navarro M., Tonholo J., Ribeiro A.S. (2012). A multielectrochromic copolymer based on pyrrole and thiophene derivatives. Sol. Energy Mater. Sol. Cells.

[18] Gadgil B., Damlin P., Ääritalo T., Kankare J., Kvarnström C. (2013). Electrosynthesis and characterization of viologen cross linked thiophene copolymer. Electrochim. Acta.

[19] Gadgil B., Damlin P., Ääritalo T., Kvarnström C. (2014). Electrosynthesis of viologen cross-linked polythiophene in ionic liquid and its electrochromic properties. Electrochim. Acta.

[20] Çamurlu P., Gültekin C., Bicil Z. (2012). Fast switching, high contrast multichromic polymers from alkyl-derivatized dithienylpyrrole and 3,4-ethylenedioxythiophene. Electrochim. Acta..

[21] Hacioglu S.O., Toksabay S., Sendur M., Toppare L. (2014). Synthesis and electrochromic properties of triphenylamine containing copolymers: Effect of π-bridge on electrochemical properties. J. Polym. Sci. Part A Polym. Chem..

[22] Wang H.M., Hsiao S.H. (2011). Enhanced redox stability and electrochromic properties of aromatic polyamides based on *N*,*N*-bis(4-carboxyphenyl)-*N*′,*N*′-bis(4-*tert*-butylphenyl)-1,4-phenylenediamine. J. Polym. Sci. Part A Polym. Chem..

[23] Atılgan N., Cihaner A., Önal A.M. (2010). Electrochromic performance and ion sensitivity of a 2,4-terthienyl based fluorescent polymer. React. Funct. Polym..

[24] Güven N., Çamurlu P. (2015). Electrosyntheses of anthracene clicked poly(thienylpyrrole)s and investigation of their electrochromic properties. Polymer.

[25] Güven N., Çamurlu P., Yücel B. (2015). Multichromic polymers based on pyrene clicked thienylpyrrole. Polym. Int..

[26] Sefer E., Bilgili H., Gultekin B., Tonga M., Koyuncu S. (2015). A narrow range multielectrochromism from 2,5-di-(2-thienyl)-1H-pyrrole polymer bearing pendant perylenediimide moiety. Dyes Pigments.

[27] Hwang J., Son J.I., Shim Y.B. (2010). Electrochromic and electrochemical properties of 3-pyridinyl and 1,10-phenanthroline bearing poly(2,5-di(2-thienyl)-1*H*-pyrrole) derivatives. Sol. Energy Mater. Sol. Cells.

[28] Turkarslan O., Ak M., Tanyeli C., Akhmedov I.M., Toppare L. (2007). Enhancing electrochromic properties of conducting polymers via copolymerization: Copolymer of 1-(4-fluorophenyl)-2,5-di(thiophen-2-yl)-1H-pyrrole with 3,4-ethylene dioxythiophene. J. Polym. Sci. Part A Polym. Chem..

[29] Wu T.Y., Chen B.K., Hao L., Lin K.F., Sun I.W. (2011). Thermophysical properties of a room temperature ionic liquid (1-methyl-3-pentyl-imidazolium hexafluorophosphate) with poly(ethylene glycol). J. Taiwan Inst. Chem. Eng..

[30] Cheng X., Zhao J., Cui C., Fu Y., Zhang X. (2012). Star-shaped conjugated systems derived from thienyl-derivatized poly(triphenylamine)s as active materials for electrochromic devices. J. Electroanal. Chem..

[31] Wu T.Y., Liao J.W., Chen C.Y. (2014). Electrochemical synthesis, characterization and electrochromic properties of indan and 1,3-benzodioxole-based poly(2,5-dithienylpyrrole) derivatives. Electrochim. Acta.

[32] Welsh D.M., Kumar A., Meijer E.W., Reynolds J.R. (1999). Enhanced contrast ratio and rapid switching in electrochromics based on poly(3,4-propylenedioxythiophene) derivatives. Adv. Mater..

[33] Ӧzkut M.İ., Atak S., Ӧnal A.M., Cihaner A. (2011). A blue to highly transmissive soluble electrochromic polymer based on poly(3,4-propylenedioxyselenophene) with a high stability and coloration efficiency. J. Mater. Chem..

[34] Hu B., Zhang Y., Lv X., Ouyang M., Fu Z., Zhang C. (2012). Electrochemical and electrochromic properties of a novel copolymer based on perylene and EDOT. Opt. Mater..

[35] Chang K.H., Wang H.P., Wu T.Y., Sun I.W. (2014). Optical and electrochromic characterizations of four 2,5-dithienylpyrrole-based conducting polymer films. Electrochim. Acta..

[36] Wu T.Y., Li W.B., Kuo C.W., Chou C.F., Liao J.W., Chen H.R., Tseng C.G. (2013). Study of Poly(methyl methacrylate)-based gel electrolyte for electrochromic device. Int. J. Electrochem. Sci..

[37] Cheng X., Zhao J., Fu Y., Cui C., Zhang X. (2013). Electrosynthesis and characterization of a multielectrochromic copolymer of tris[4-(2-thienyl)phenyl]amine with 3,4-ethylenedioxythiophene. J. Electrochem. Soc..

[38] Xu C., Zhao J., Cui C., Wang M., Kong Y., Zhang X. (2012). Triphenylamine-based multielectrochromic material and its neutral green electrochromic devices. J. Electroanal. Chem..

[39] Cihaner A., Algı F. (2008). An electrochromic and fluorescent polymer based on 1-(1-naphthyl)-2,5-di-2-thienyl-1H-pyrrole. J. Electroanal. Chem..

[40] Bicil Z., Camurlu P., Yucel B., Becer B. (2013). Multichromic, ferrocene clicked poly(2,5-dithienylpyrrole)s. J. Polym. Res..

[41] Camurlu P., Gültekin C. (2012). A comprehensive study on utilization of N-substituted poly(2,5-dithienylpyrrole) derivatives in electrochromic devices. Sol. Energy Mater. Sol. Cells.

[42] Chen S., Gao Q., Zhao J., Cui C., Yang W., Zhang X. (2012). Electrochemical synthesis and characterization of a new electrochromic copolymer based on 2,2′-bithiophene and tris[4-(2-thienyl)phenyl]amine. Int. J. Electrochem. Sci..

[43] Kuo C.W., Hsieh T.H., Hsieh C.K., Liao J.W., Wu T.Y. (2014). Electrosynthesis and characterization of four electrochromic polymers based on carbazole and indole-6-carboxylic acid and their applications in high-contrast electrochromic devices. J. Electrochem. Soc..

[44] Ouyang M., Fu Z., Lv X., Hu B., Wang P., Huang S., Dai Y., Zhang C. (2013). A Multichromic copolymer based on 4-(9H-carbazol-9-yl)-N,N-diphenylaniline and 3,4-ethylenedioxythiophene prepared via electrocopolymerization. J. Electrochem. Soc..

